# 

**Published:** 1903-03-07

**Authors:** 


**MISSING.**

Bertram or Bert Miles, age 53. Can any House Surgeon or Head Nurse say if such a person has been an in or out patient in any hospital in or around London or elsewhere within the last three years, if so, is he known to be alive or dead? A reply through "The Hospital Office" will oblige his aged and widowed mother. (2751)

### PUBLISHER'S NOTICE.

To the Subscribers and Readers of "The Hospital."

### THE PRICE OF "THE HOSPITAL."

The circulation of The Hospital is now so large as to make it essential that the price shall approximate more closely to the actual cost of production than it has done in the past. It is therefore found necessary, after full consideration, to increase the price of this Journal **from 2d. to 3d. per copy.** This change in price will take effect from the first number of Volume XXXIV., which will be issued on Saturday, April 4th, 1903. The terms of subscription from that date will be as follows:—

| | Great Britain. Post Free. | Foreign and Colonial. Post Free. |
|---|---|---|
| For One Year ... | 15s. 0d. | 19s. 6d. |
| " Six Months ... | 7s. 6d. | 10s. 0d. |
| " Three Months ... | 3s. 9d. | 5s. 0d. |

Monthly Parts.

| | Post Free to any Part of the World. |
|---|---|
| For One Year ... ... ... | 15s. 6d. |
| " Six Months ... ... ... | 7s. 9d. |
| " Three Months ... ... ... | 4s. 0d. |

N.B.—Subscribers who have already paid their subscriptions will continue to receive The Hospital as issued at the old rates until such subscriptions expire.

**All new Subscriptions commencing on and after March 7th, 1903, will be charged at the above rates.**

Publishing and Editorial Offices, "The Hospital" Buildings, 28 & 29 Southampton Street, Strand, London, W.C.

### The Student of Medicine.

### APPOINTMENTS.

**John D'Ewart**, M.R.C.S., L.R.C.P., appointed Third Resident Assistant Medical Officer at the Crumpsall Workhouse, Manchester. **W. d'Este Emery**, M.D., B.Sc.Lond., appointed Clinical Pathologist to King's College Hospital. **R. Bruce Ferguson**, M.A., M.D., B.C.Cantab., M.R.C.S., L.R.C.P., appointed Medical Officer and Public Vaccinator for the Seventh (New Southgate) District of the Barnet Union. **J. H. Fletcher**, M.R.C.S. L.R.C.P.Lond., reappointed Medical Officer of Health for the Ince District Council. **Francis D. Grayson**, M.R.C.S.Eng., L.S.A., appointed Medical Officer of Health for the Rochford Rural District. **Hy. Hutchinson**, L.R.C.P.I., L.F.P.S.Glasg., L.S.A.Lond., appointed Surgeon to the Metropolitan District Railway Provident Society for the suburbs of East Ham, Upton, and Manor Park. **Edgar Reginald Ivatts**, M.R.C.S., L.R.C.P.Lond., appointed Medical Officer for the Mellor District of the Blackburn Union. **John Jarvis**, M.R.C.S., appointed Surgeon to the We tern Dispensary, Bath. **E. Lewys-Lloyd**, M.R.C.S., L.R.C.P.Lond., appointed Medical Officer of Health for the Towyn District. **C. Peddie**, M.A., L.R.C.P., appointed Medical Officer for the Union and Public Vaccinator for the Brindle District of Chorley. **E. H. Roberts**, M.R.C.S., L.R.C.P., appointed District Medical Officer of the Anglesey Union. **T. E. Sandall**, M.B., B.C.Cantab., M.R.C.S., L.R.C.P., appointed Certifying Factory Surgeon for the Alford District of the County of Lincoln. **Edmund Ivens Spriggs**, M.D.Lond., M.R.C.P.Lond., appointed Physician to Out-patients to the Victoria Hospital for Children, and Physician to Out-patients to the City of London Hospital for Diseases of the Chest, Victoria Park, E. **Tom Walker**, M R.C.S., L.R.C.P., appointed Second Resident Assis'ant Medical Officer at the Crumpsall Workhouse, Manchester. **C. Gordon Watson**, F.R.C.S.Eng., appointed Casualty Officer, Registrar, and Pathologist to the Metropolitan Hospital. **Arthur G. Wilkins**, M.B., Ch.B.Vict., appointed Medical Officer to the West Ward Union of Westmorland, and Public Vaccinator and Certifying Factory Surgeon for the Hartsop and Patterdale District. **J. O. Williams**, M.R.C.S., L.R.C.P.Lond., appointed District Medical Officer of the Dolgelly Union. **C. H. Wood**, M.R.C.S.Eng., M.R.C.P.Lond., appointed Second House Physician to the City of London Hospital for Diseases of the Chest, Victoria Park.

Manchester Royal Infirmary.—The following appointments have been made:—Resident Medical Officer: **H. J. Crompton**, M.B., Ch. Vict. Assistant Medical Officer: **W. Riddell Matthews**, M.B., C.M.Aberd. House Surgeons: **E. B. Leech**, M.R.C.S., L.R.C.P.; **W. Stansfield**, M.R.C.S., L R.C.P.; and **C. Mackey**, M.B., Ch.B.Vict. House Physicians: **H. B. Wyman**, L.R C.P., L.R.C.S.Edin., and **J. S. Smith**, M.R.C.S., L.R.C.P.

St. Thomas's Hospital.—The following gentlemen have been selected as House Officers, from Tuesday, March 3rd, 1903:—House Physicians: **C. N. Sears**, L.R.C.P., M.R.C.S.; **A. E. Boycott**, M.A., M.B., B.Ch.Oxon., B.Sc.Oxon.; **W. H. Harwood Yarred**, B Sc.Lond., L.R.C.P., M.R.C.S. (*Extension*); **A. Mavrogordato**, B.A.Oxon., L.R.C.P., M.R.C.S. (*Extension*). Assistant House Physicians: **O. Hildesheim**, B.A., M.B., B.Ch.Oxon., L.R.C.P., M.R.C.S.; **H. W. Sexton**, L.R.C.P., M.R.C.S. House Surgeons: **J. W. Rob**, B.A., M.B., B.C.Cantab.; **T. B. Henderson**, M.A., M.B., B.Ch.Oxon.; **J. P. Hedley**, M.A., M.B., B.C.Cantab.; **A. B. Bradford**, M.B., B.S.Durh., L.R.C.P., M.R.C.S.; Assistant House Surgeons: **J. E. Adams**, L.R.C.P., M.R.C.S.; **H. Upcott**, L.R.C.P., M.R.C.S.; **C. Wheen**, B.A.Oxon., L.R.C.P., M.R.C.S.; **N. Carpmael**, L R.C.P., M.R.C.S. Obstetric House Physicians: (*Senior*) **G. A. C. Shipman**, M.A., M.B., B.C.Cantab., L.R.C.P., M.R.C.S.; (*Junior*) **W. M. G. Glanville**, B.A., M.B., B.Ch.Oxon. Ophthalmic House Surgeons: (*Senior*) **A. E. A. Loosely**, B.A.Oxon., L.R.C.P., M.R.C.S.; (*Junior*) **A. C. Hudson**, M.A., M.B., B.C.Cantab. Clinical Assistants: (Throat) **H. S. D. Browne**, B.A.Cantab., L.R.C.P., M.R.C S. (*Extension*); **R. E. H. Leach**, B.A.Oxon., L R.C.P., M.R.C.S. (Skin) **T. Guthrie**, B.A.Cantab., L.R.C.P., M.R.C.S; **W. M. Strong**, M.A., B.C.Cantab., L.R.C.P., M.R.C.S. (Ear) **B. S. Jones**, L.R.C.P., M.R.C.S. (*Extension*); **A. Bevan**, M.B.Lond., L.R.C.P., M.R.C.S. Clinical Assistants: (Electrical Department) **W. M. Strong**, M.A., B.C Cantab., L.R.C.P., M.R.C.S. (X-Ray Department) **R. Small**, L.R.C.P., M.R.C.S. (*Extension*).

### PUBLISHER'S NOTICE.

TO SUBSCRIBERS AND READERS OF "THE HOSPITAL."

### THE PRICE OF "THE HOSPITAL."

The circulation of The Hospital is now so large as to make it essential that the price shall approximate more closely to the actual cost of production than it has done in the past. It is therefore found necessary, after full consideration, to increase the price of this Journal **from 2d. to 3d. per copy**. This change in price will take effect from the first number of Volume XXXIV., which will be issued on Saturday, April 4th, 1903. The terms of subscription from that date will be as follows:—

| | Great Britain. (Post free.) | Foreign and Colonial. (Post free.) |
|---|---|---|
| For one year ... ... | 15s. 0d. | 19s. 6d. |
| For six months... ... | 7s. 6d. | 10s. 0d. |
| For three months ... | 3s. 9d. | 5s. 0d. |

| Monthly Parts. | Post free to any part of the World. |
|---|---|
| For one year ... ... ... | 15s. 6d. |
| For six months ... ... ... | 7s. 9d. |
| For three months ... ... ... | 4s. 0d. |

N.B.—Subscribers who have already paid their subscriptions will continue to receive The Hospital as issued at the old rates until such subscriptions expire.

**All new Subscriptions commencing on and after March 7, 1903, will be charged at the above Rates.**

Publishing and Editorial Offices, "The Hospital" Buildings, 28 & 29 Southampton Street, Strand, London, W.C.

### "Sir Henry Burdett's monumental work on Hospitals."

*The Times.*

PROSPECTUS AND ORDER FORM POST FREE ON APPLICATION.

THE SCIENTIFIC PRESS, LTD.,
28 & 29 Southampton Street, Strand, London, W.C.

